# Mild hyperbaric oxygen does not attenuate mitochondrial decrease induced by detraining in mice

**DOI:** 10.1016/j.bbrep.2025.102341

**Published:** 2025-11-06

**Authors:** Ai Takemura, Tatsuro Egawa, Kazuki Uemichi, Ryota Iyama, Haiyu Zhao, Mayuko Oba, Tatsuya Hayashi, Satoshi Fujita

**Affiliations:** a Ritsumeikan Global Innovation Research Organization, Ritsumeikan University, Shiga, Japan; b Graduate School of Human and Environmental Studies, Kyoto University, Kyoto, Japan; c Faculty of Sport and Health Science, Ritsumeikan University, Shiga, Japan

**Keywords:** Mild hyperbaric oxygen, Detraining, Mitochondria, Voluntary wheel running

## Abstract

Mild hyperbaric oxygen (MHO) attenuates the muscle atrophy caused by muscle disuse. Training cessation results in the partial or complete loss of training-induced adaptations, including mitochondrial enzyme activities. The present study aimed to determine whether exposure to MHO after running training attenuated negative adaptation induced by detraining. We allocated eight-week-old mice into training (Tr), detraining after the training period (DeTr), and detraining + mild hyperbaric oxygen (DeTr + MHO, 1.3 atm absolute with 38 % oxygen) groups. Mice underwent voluntary wheel running for four weeks, followed by a two-week detraining period under normal or MHO conditions. The soleus muscle weight (mg/g BW) decreased by approximately 30 % in the DeTr and DeTr + MHO groups compared to the Tr group (*P* < 0.001 and 0.01, respectively). Citrate synthase (CS) activity, the expression of mitochondrial complex IV, antioxidant-related proteins, catalase, and heme oxygenase 1 (HO-1), decreased in the DeTr and DeTr + MHO group compared to the Tr group (*P* < 0.05). In summary, MHO did not attenuate the detraining-induced decrease in soleus muscle weight relative to body weight, mitochondrial enzyme activity, protein, or antioxidant protein expression level in the plantaris muscle after a four-week training period using voluntary wheel running in mice.

## Highlights

• Detraining reduced soleus weight and plantaris mitochondrial enzyme activity.
• MHO did not prevent the decrease in soleus weight after detraining.
• MHO failed to prevent mitochondrial and antioxidant protein loss.

## 1 Introduction

Athletes and physically active individuals may suspend training because of illness, injury, or other constraints. This detraining progressively reverses exercise-induced adaptations, causing declines in skeletal muscle mass, cardiorespiratory fitness (e.g., maximal oxygen uptake and oxygen pulse), mitochondrial enzyme activity, antioxidant defenses, and endurance performance [[1], [2], [3], [4], [5]]. It is essential to develop strategies to prevent the negative adaptations associated with detraining, thereby enhancing the competitiveness of athletes. A previous studies showed that detraining reduced peroxisome proliferator-activated receptor-γ coactivator-1α (Pgc-1α, a key protein of mitochondrial biogenesis) mRNA, citrate synthase (CS) activity, cytochrome *c* oxidase subunit IV protein expression levels and antioxidant system [[5], [6], [7]], and increased catabolism-related proteins expression, such as atrogin-1 and MuRF1, in the skeletal muscle [8]. Mitochondrial dynamics, including mitochondrial fission and fusion, regulate mitochondrial homeostasis and quality [9]. A previous study showed that detraining after treadmill running decreased optic atrophy 1 (Opa1), a protein involved in mitochondrial fusion [6].

Hyperbaric oxygen therapy, typically administered at 2–3 atm absolute (ATA) with 100 % oxygen, is commonly used in the treatment of conditions such as chronic wounds, decompression sickness, and carbon monoxide poisoning. Previous studies have shown that exposure to hyperbaric oxygen therapy (HBOT) condition increases antioxidant enzymes, including catalase, superoxide dismutase, and glutathione peroxidase, thereby reducing oxidative stress [10,11]. Although effective, HBOT is also associated with side effects such as barotrauma and oxygen toxicity [12]. The main risk factors for barotrauma and oxygen toxicity are compression rate and increased treatment pressure, respectively [12]. In contrast to HBOT, mild hyperbaric oxygen (MHO) is an environment of 1.25–1.30 ATA with 36–40 % oxygen. Because of the lower pressure and oxygen concentration compared with HBOT, MHO is considered to present a lower risk of side effects [13]. Although HBOT has been reported to enhance antioxidant capacity, it remains unclear whether MHO exerts similar effects. MHO has been reported to ameliorate muscle atrophy and increase oxidative metabolism of skeletal muscle in models of muscle disuse, diabetes, and metabolic syndrome in rodents [[14], [15], [16]]. In a rat model of hindlimb unloading–induced muscle atrophy, exposure to MHO for four weeks elevated Pgc-1α mRNA levels while reducing the expression of forkhead box O1 (FOXO1), an atrophy-related gene, in the soleus muscle [16]. However, the effects of exposure to MHO on antioxidant capacity and on the negative adaptations induced by detraining remain unclear.

Previous studies have also shown that mitochondrial adaptations induced by treadmill endurance running were more pronounced in fast-twitch muscles than in slow-twitch muscles [17,18]. Previous studies have shown that MHO attenuates the reduction of mitochondrial proteins and enzyme activity in the soleus, plantaris, extensor digitorum longus muscle [[14], [15], [16],^19^], suggesting that MHO may increase the oxidative capacity regardless of muscle type, including both slow-twitch and fast-twitch fibers. With respect to muscle mass, treadmill training increased the weight of both the soleus and plantaris muscles by a similar extent [18]. However, findings on the effects of MHO are inconsistent: one study showed that it attenuated plantaris atrophy but not soleus atrophy in a casting immobilization model [19], whereas another reported that MHO given before and after hindlimb unloading inhibited the reduction in soleus weight [16]. Thus, although MHO has demonstrated beneficial effects on muscle weight, it is still uncertain whether these adaptations occur preferentially in specific muscle fiber types.

The present study aimed to determine whether exposure to MHO during detraining after the training period, using voluntary wheel running, ameliorates the muscle mitochondrial negative adaptation that occurs during detraining. We hypothesized that exposure to MHO would alleviate the reduction in muscle mitochondria-related protein levels induced by detraining by inhibiting the catabolic signaling pathway and increasing PGC-1α expression levels.

## 2 Materials and methods

### 2.1 Experimental animals and treatments

Eight-week-old male ICR mice were randomly assigned to three groups: training (Tr, n = 7), detraining (DeTr, n = 7), and detraining + mild hyperbaric oxygen (DeTr + MHO, n = 7). All animals had free access to food and water. The housing environment was maintained under controlled conditions (22 ± 2 °C, 45–55 % relative humidity) with a 12-h light/dark cycle (lights off from 20:00 to 08:00). Experimental procedures were approved by the Kyoto University Animal Experiment Committee (no. 24-A-1) and followed institutional guidelines for animal care and use.

All mice were individually housed under one ATA with 20.9 % oxygen (normobaric conditions) in a standard cage with voluntary access to resistance-free running wheels (K3570 and K3251, Bio-Serv, NJ, USA) for four weeks. The age of training was standardized to minimize the variability in the training effect due to age. The soleus and plantaris muscles of the Tr group were excised 24 h after the removal of the running wheel under anesthesia, quickly frozen in liquid nitrogen, and stored at −80 °C until analysis. After the training period, mice in the DeTr and DeTr + MHO groups were housed sedentary in a standard cage without a running wheel for 2 weeks, the detraining period. During the detraining period, mice in the DeTr + MHO groups were exposed to 1.3 ATA with 38 % oxygen for 6 h/day using a chamber for 2 weeks. Following the detraining period, mice in the DeTr and DeTr + MHO groups were anesthetized, and the soleus and plantaris muscles were excised 24 h after the final MHO exposure, immediately frozen in liquid nitrogen, and preserved at −80 °C for later analysis.

### 2.2 Western blotting

The left soleus and plantaris muscles were homogenized 20 times (vol/wt) using a bead crusher (μT-12, TAITEC, Koshigaya, Japan) in radioimmunoprecipitation assay buffer (20–188, Millipore, MA, USA) containing protease (1183617001, Complete Mini EDTA-free, Roche Life Science, Indianapolis, IN, USA) and phosphatase inhibitors (04906837001, PhosSTOP, Roche Life Science). Homogenates were incubated on ice with rotation for 60 min and centrifuged at 1500×*g* for 20 min at 4 °C. Protein content was determined using the TaKaRa BCA Protein Assay Kit (TaKaRa BIO Inc., Kusatsu, Japan). Equal amounts of protein (10 μg) were subjected to sodium dodecyl sulfate–polyacrylamide gel electrophoresis (SDS–PAGE) at 200 V. Proteins were subsequently transferred onto polyvinylidene difluoride (PVDF) membranes. The membranes were blocked for 5 min at 22 °C with EveryBlot Blocking Buffer (#12010020, Bio-Rad Laboratories, CA, USA), followed by overnight incubation at 4 °C with the following primary antibodies: atrogin-1 (ab168372, Abcam, Cambridge, UK), muscle RING finger protein-1 (MuRF-1, ab172479, Abcam), oxidative phosphorylation (OXPHOS, ab110413, Abcam), PGC-1α (516557, Millipore), fission, mitochondrial 1 (Fis1, ab96764, Abcam), phospho-Drp1 (Ser616, 3455S, CST), total-dynamin-related protein 1 (Drp1, ab56788, Abcam, Cambridge, UK), Mitofusin2 (Mfn2, ab124773, Abcam), Opa1 (612606, BD Biosciences, NJ, USA), catalase (sc-271803, Santa Cruz Biotechnology, TX, USA), and heme oxygenase (HO-1, 70081, CST). Membranes were then incubated for 60 min at 22 °C with the following secondary antibodies: anti-rabbit IgG (HRP-linked antibody, #7074, CST) and anti-mouse IgG (HRP-linked antibody, #7076, CST).

Proteins were visualized using Immobilon Forte Western HRP substrate (WBLUF0100, Millipore) and captured with a FUSION Chemiluminescence Imaging System (M&S Instruments, Osaka, Japan). Densitometric analysis of band intensities was performed with ImageJ software (NIH, Bethesda, MD, USA). Ponceau-S staining was employed to verify equal protein loading.

### 2.3 Enzyme activity assays

Whole right soleus and plantaris muscles were homogenized in 100-fold volumes (vol/wt) of phosphate buffer (100 mM, pH 7.6) with a bead crusher (μT-12, TAITEC, Koshigaya, Japan). After two cycles of freeze–thawing in liquid nitrogen, the homogenates were centrifuged at 1000×*g* for 10 min at 4 °C, and the supernatants were obtained for determination of maximal CS and cytochrome *c* oxidase (COX) activities as described previously [20,21]. Enzyme activities were normalized to protein concentration, which was determined using the BCA assay (TaKaRa BIO Inc.).

### 2.4 Statistical analysis

Data from all animals were used for outlier detection with Grubbs’ test, and any values identified as outliers were excluded from further statistical analyses. Results are reported as mean values ± standard deviation (SD). Comparisons among Tr, DeTr, and DeTr + MHO groups were performed using one-way analysis of variance, followed by Tukey–Kramer multiple-comparison tests. Statistical analyses were carried out using GraphPad Prism (version 9.0; GraphPad Software, La Jolla, CA, USA). Statistical significance was set at P < 0.05.

## 3 Results

### 3.1 Muscle weights

**Fig. 1:**
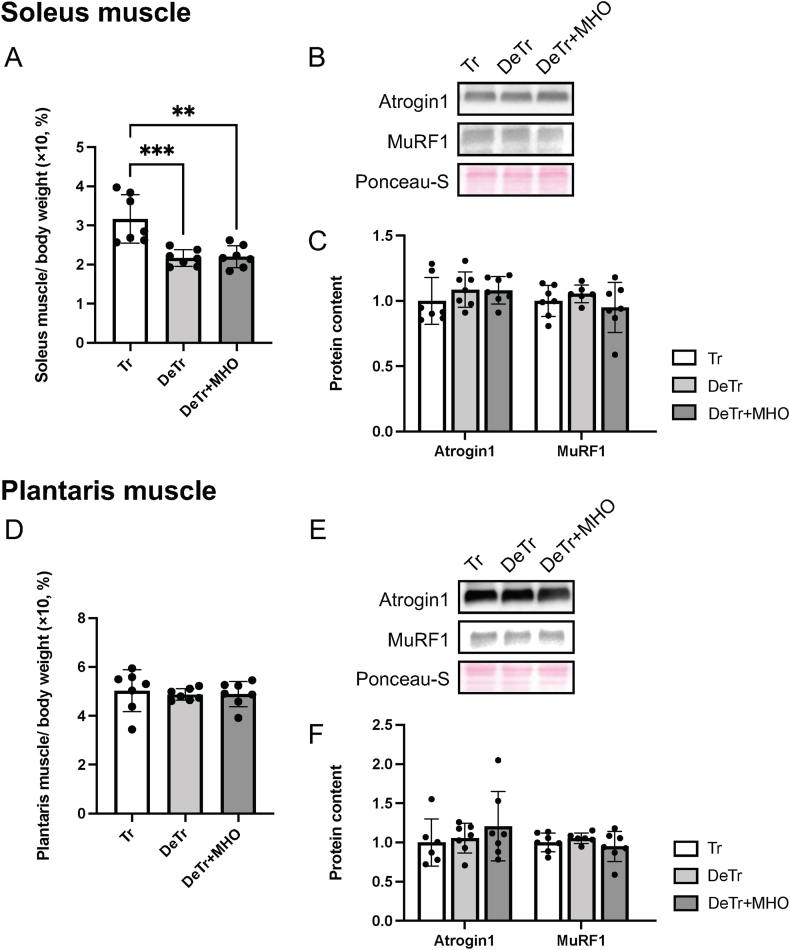
Effects of mild hyperbaric oxygen (MHO) on the muscle weight (%body weight, A and D), representative western blots (B and E), and expression levels of Atrogin-1 and MuRF1 (C and F) in the soleus and plantaris muscles in detraining mice. Values are presented as mean ± SD (n = 7). ∗∗*P* < 0.01 and ∗∗∗*P* < 0.001. Tr, training; DeTr, detraining; DeTr + MHO, detraining with mild hyperbaric oxygen.

The soleus muscle weight (mg/g BW) was lower in the DeTr and DeTr + MHO group than in the Tr group (*P* < 0.001 and 0.01, repetitively) (Fig. 1A). There was no difference in plantaris muscle weight (mg/g BW) among the three groups (Fig. 1D).

### 3.2 Muscle atrophy-related proteins

There was no difference in the Atrogin-1 and MuRF1 expression levels in the soleus (Fig. 1B and C) and plantaris muscles (Fig. 1E and F) among the three groups.

### 3.3 Mitochondria-related proteins and enzyme activity

**Fig. 2:**
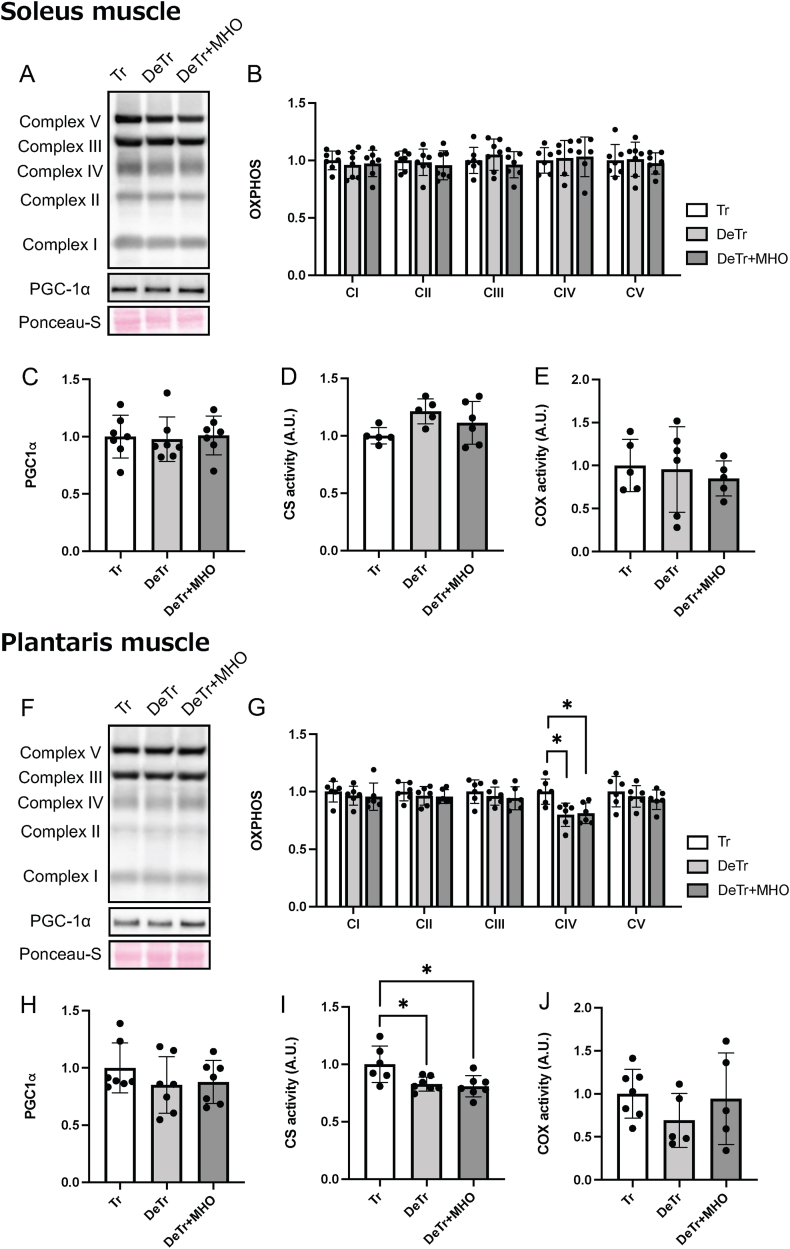
Representative western blots (A and F), the expression levels of mitochondrial proteins (complex I; NDUFB8, complex Ⅱ; SDHB, complex Ⅲ; UQCRC2, complex Ⅳ; MTCO1, and complex Ⅴ; ATP5A, B and G), PGC-1α protein levels (C and H), and CS (D and I) and COX (E and J) activities in the soleus and plantaris muscles in mice. Values are presented as mean ± SD (n = 7). ∗*P* < 0.05. Tr, training; DeTr, detraining; DeTr + MHO, detraining with mild hyperbaric oxygen.

There were no differences in the expression levels of mitochondrial proteins (complex I; NDUFB8, complex Ⅱ; SDHB, complex Ⅲ; UQCRC2, complex Ⅳ; MTCO1, complex Ⅴ; ATP5A, Fig. 2A and B), PGC-1α protein (Fig. 2A and C), or CS and COX activities (Fig. 2D and E) among the three groups in the soleus muscle. The expression of complex IV protein was decreased in the DeTr and DeTr + MHO groups compared to the Tr group in the plantaris muscle (Fig. 2F and G). There were no differences in the expression levels of complex I, II, III, and V proteins, PGC-1α protein, or COX activity among the three groups in the plantaris muscle (Fig. 2F–H and J). The CS activity of the plantaris muscle was lower in the DeTr and DeTr + MHO groups than in the Tr group (*P* < 0.05) (Fig. 2I).

**Fig. 3:**
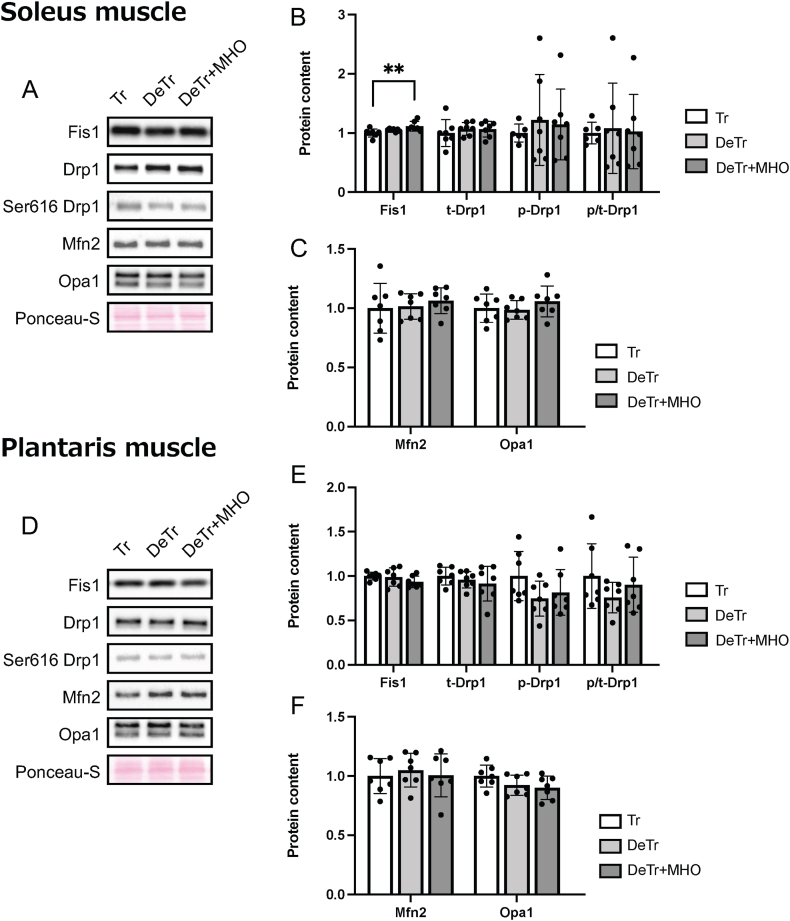
Representative western blots (A and D) and the protein levels of mitochondrial fission (Fis1, Drp1, and phospho-Drp1, B and E) and fusion (Mfn2 and Opa1, C and F) in the soleus and plantaris muscles in mice. Values are presented as mean ± SD (n = 7). ∗∗*P* < 0.01. Tr, training; DeTr, detraining; DeTr + MHO, detraining with mild hyperbaric oxygen.

In the mitochondrial fission-related proteins, the expression level of Fis1 was increased in the DeTr + MHO compared to the Tr group in the soleus muscle (Fig. 3A and B). There were no differences in the levels of phospho- and total-Drp1 expression in the soleus and plantaris muscles and the level of Fis1 expression in the plantaris muscle among the three groups (Fig. 3D and E). In the mitochondrial fusion proteins, there were no differences in the levels of Mfn2 and Opa1 protein expression among the three groups in the soleus and plantaris muscles (Fig. 3C and F).

### 3.4 Antioxidant-related proteins

**Fig. 4:**
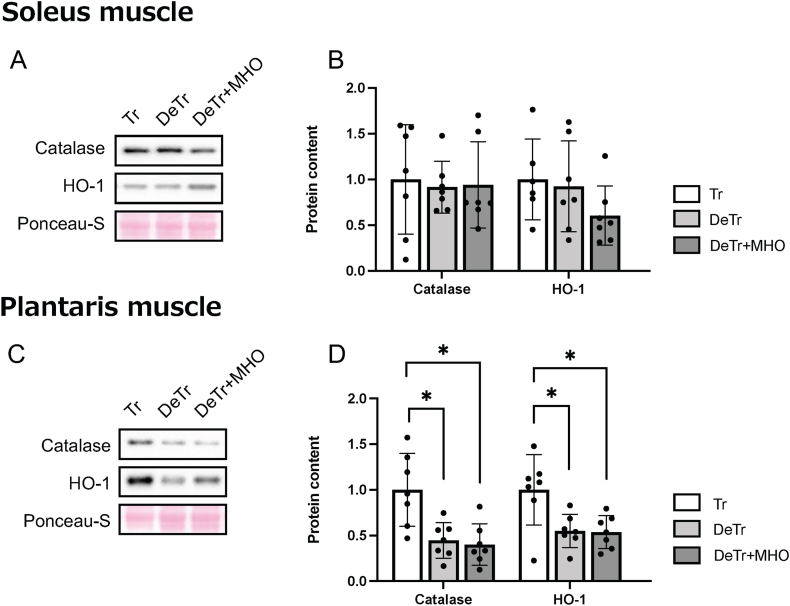
Representative western blots (A and C) and the antioxidant protein (catalase and HO-1) levels (B and D) in the soleus and plantaris muscles in mice. Values are presented as mean ± SD (n = 7). ∗*P* < 0.05. Tr, training; DeTr, detraining; DeTr + MHO, detraining with mild hyperbaric oxygen.

There were no differences in the catalase and HO-1 protein expression levels in the soleus muscle among the three groups (Fig. 4A and B). The expression levels of catalase and HO-1 in the plantaris muscle were lower in the DeTr and DeTr + MHO group than in the Tr group (*P* < 0.05) (Fig. 4C and D).

## 4 Discussion

We investigated the effects of MHO on the muscle mitochondrial enzyme activity and protein levels after detraining in mice. Detraining reduced soleus muscle weight and CS activity, as well as the expression of mitochondrial complex IV, in the plantaris muscle compared to trained mice. However, MHO during the detraining period did not alleviate these negative adaptations induced by detraining in mice.

Although the present study did not include a wheel-running only control group, prior work provides a clear expectation for the effects of voluntary wheel running alone in healthy mice. In mouse models, four weeks of voluntary wheel running generally does not produce robust whole-muscle hypertrophy in mixed or predominantly fast-twitch muscles: several studies report no significant change in tibialis anterior (TA), plantaris, or gastrocnemius muscle weights after this training period [22,23]. By contrast, fiber-level analyses indicate selective remodeling within the gastrocnemius, where the cross-sectional area of type I fibers increased following the same intervention [23]. Concordantly, slow-twitch soleus appears relatively favored: one study observed up to 13 % increase in soleus mass after four weeks of wheel running, although the difference versus sedentary controls did not reach statistical significance [24]. Further, the same training was associated with approximately 9 % decreases in fast-twitch muscles (extensor digitorum longus and TA) alongside the ∼13 % increase in soleus mass [24]. Collectively, in healthy mice, four weeks of wheel running yields little or no whole-muscle hypertrophy in TA, plantaris, or gastrocnemius [22,23], but promotes modest, fiber-type–biased remodeling, enlarged type I fibers and a trend toward increased soleus mass [23,24]. These muscle-specific patterns (stable plantaris vs. upward-trending soleus) suggest greater responsiveness of slow-than fast-twitch muscles to submaximal habitual loading.

Previous studies have shown that voluntary wheel running training for four weeks increases mitochondrial complex protein expression and enzyme activity in mice [22,25]. In addition, the training reduced the rate of damaged mitochondria via restoration of mitochondrial dynamics, including increased mitochondrial fusion and fission [22,25]. Taken together, these finding suggest that wheel running training may enhance mitochondrial content and quality.

The present study showed that detraining for two weeks after four weeks of training reduced the soleus muscle weight relative to body weight compared to trained mice. However, there were no differences between groups in the plantaris muscle relative to body weight. A previous study demonstrated that detraining for two weeks after four weeks of treadmill running did not result in weight loss in the plantaris muscle [6]. In a previous study, the weight of the soleus muscle relative to body weight on the detrained day 14 after three weeks of voluntary treadmill training was reduced by approximately 11 % compared to the detrained day 1, whereas the weight of the plantaris muscle relative to body weight was reduced by approximately 4 % compared to the detrained day 1 [26]. These results suggest that the soleus muscle is more susceptible than the plantaris muscle to detraining. In this study, MHO did not attenuate the loss of soleus muscle weight induced by detraining. MHO at 1.25–1.30 ATA with 36–40 % oxygen, when administered for at least four weeks, has been shown to attenuate skeletal muscle atrophy in rat models of muscle disuse, diabetes, and metabolic syndrome. [[14], [15], [16]]. In a hindlimb unloading model of muscle atrophy, combined pre- and post-conditioning with MHO reduced FOXO1 mRNA expression [16], suggesting that MHO may attenuate muscle catabolism by inhibiting catabolic signaling. On the contrary, there was no significant difference in catabolic signaling, as indicated by MuRF1 and Atrogin-1, between the Tr group and the DeTr group in this study. MHO may not attenuate the muscle weight loss induced by detraining due to the normal expression levels of catabolic proteins. Muscle atrophy is induced by detraining and muscle disuse via reduction of anabolic systems [3,27]. A previous study showed that MHO did not alter the phosphorylation ratio of anabolic signaling proteins, such as p70 ribosomal S6 kinase, 4E-binding protein 1, and ribosomal protein S6, during the recovery period after casting immobilization in rats [19]. These findings suggest that MHO may not affect anabolic signaling in the detraining condition.

In this study, detraining for two weeks resulted in reduced mitochondrial CS activity and complex IV in the plantaris muscle compared to trained mice. Similar to this study, a previous investigation demonstrated that detraining for two weeks after four weeks of treadmill running resulted in reduced CS activity and decreased cytochrome *c* oxidase subunit IV protein expression levels in the plantaris muscle [6]. In the present study, exposure to MHO did not attenuate the detraining-induced reductions in mitochondrial CS activity and complex IV protein levels. While CS activity was reduced by detraining, COX activity remained unchanged, suggesting that COX activity normalized to protein content may not be altered by detraining. However, COX activity measured in homogenates under artificial assay conditions provides information on the maximal catalytic capacity of complex IV but does not allow for the evaluation of respiratory coupling [21]. Therefore, it remains unclear whether MHO affects mitochondrial respiratory function during detraining. A previous study demonstrated that when rats were exposed to MHO (1.25 ATA, 36 % oxygen) both before and after hindlimb unloading for a total duration of four weeks, increases in Pgc-1α mRNA expression and succinate dehydrogenase (SDH) activity were observed [16]. In contrast, limiting MHO treatment to only the two-week recovery period after unloading did not prevent the declines in these measures [16]. These studies suggest that two weeks of MHO exposure is too short to maintain mitochondrial content in the disuse condition. In terms of mitochondrial dynamics, a previous study demonstrated that detraining after treadmill running resulted in decreased Opa1, a mitochondrial fusion-related protein [6]. In this study, there were no significant differences between the Tr and DeTr groups in the expression of proteins related to mitochondrial dynamics, fusion, and fission. The reduced Opa1 expression induced by detraining in the previous study [6] may be a specific adaptation after treadmill running at a medium to high intensity, as opposed to detraining after voluntary wheel running at low intensity. In this study, the mitochondrial content after detraining using wheel running may have been induced by other signaling pathways, such as oxidative stress [28,29] or inflammation [30], rather than mitochondrial dynamics, fusion, and fission. The protein level of Fis1, a mitochondrial fission protein, increased in the DeTr + MHO group compared to the Tr group. There were no differences in Fis1 expression between the Tr and DeTr groups, suggesting that MHO may be responsible for the increased Fis1 levels. These findings indicate that MHO may influence mitochondrial dynamics. Further studies are needed to clarify the effect of MHO on mitochondrial dynamics.

A previous study showed that voluntary wheel running training for four weeks reduced high levels of oxygen stress markers, mitochondrial 4-hydroxynonenal and protein carbonyl levels [25]. Another study showed that the training increased superoxide dismutase (SOD), which are key antioxidant enzymes [31]. These results suggest that wheel running may increase antioxidant capacity and decrease oxidative stress in the present study. The present study demonstrated that detraining for two weeks resulted in reduced expression levels of the antioxidant proteins catalase and HO-1, compared to trained mice, and concurrently decreased mitochondrial enzyme activity. Exercise training causes an adaptation in the antioxidant system, although the adaptation is reversed by detraining [5]. Antioxidant genes such as catalase and HO-1 scavenge ROS and maintain intracellular redox homeostasis [32]. The reduction in the antioxidant system induced by detraining was not alleviated by exposure to MHO. A previous study showed that an acute hyperbaric oxygen treatment, conducted in an environment of 2.4 ATA and 100 % oxygen for 1 h, induces the expression of antioxidant genes, including HO-1 mRNA, in the human microvascular endothelial cell line [33]. These studies, including this one, suggest that MHO at 1.3 ATA and 38 % oxygen is insufficient to enhance the antioxidant system for two weeks in the detraining condition.

Although the present study was conducted using only male mice, the potential influence of sex differences on mitochondrial and antioxidant adaptations to exercise cannot be excluded. Previous studies have shown that females exhibit higher cytochrome *c* oxidase protein content and antioxidant defenses compared with males in sedentary rodents, largely due to estrogen action [[34], [35], [36]]. Women have been shown to display higher fat oxidation than men during acute endurance exercise, as reflected by a lower respiratory exchange ratio and differences in sex hormones [[37], [38], [39]]. According to a previous study, the beneficial antioxidative adaptations induced by training were maintained longer in males than in female rats during detraining [31]. Therefore, while the present findings provide new insight into the effects of MHO in male mice, future studies incorporating both sexes will be required to clarify whether MHO exerts sex-specific effects on skeletal muscle metabolism.

The present study has several limitations. First, we did not include a sedentary control group, which limits our ability to determine the extent of muscle adaptations induced by training. Second, mitochondrial oxygen consumption rate (OCR), a widely accepted indicator of mitochondrial function, was not measured in the present study. Whether MHO alters mitochondrial respiratory capacity during detraining remains to be clarified, and future studies should directly assess OCR in fresh muscle samples. Third, we were unable to measure the ratio of muscle fiber types in the soleus and plantaris muscles. In this study, different adaptations were induced by detraining in the two muscles, suggesting that distinct adaptations may occur between the fiber types. Therefore, the effects of detraining and/or MHO on each fiber type remain unclear.

## 5 Conclusions

Detraining after a training period using voluntary wheel running resulted in a reduction in soleus muscle weight (% body weight). Mitochondrial enzyme activity and antioxidant protein expression in the plantaris muscle were also decreased by detraining. MHO under 1.3 ATA with 38 % oxygen did not alleviate the reduced mitochondrial enzyme activity in the plantaris muscle of detrained mice compared to trained mice.

## CRediT authorship contribution statement

**Ai Takemura:** Funding acquisition, Investigation, Methodology, Project administration, Writing – original draft. **Tatsuro Egawa:** Investigation, Methodology, Writing – review & editing. **Kazuki Uemichi:** Investigation, Methodology, Writing – review & editing. **Ryota Iyama:** Methodology, Writing – review & editing. **Haiyu Zhao:** Methodology, Writing – review & editing. **Mayuko Oba:** Methodology, Writing – review & editing. **Tatsuya Hayashi:** Project administration, Writing – review & editing. **Satoshi Fujita:** Project administration, Writing – review & editing.

## Declaration of competing interest

The authors declare the following financial interests/personal relationships which may be considered as potential competing interests:Ai Takemura reports financial support was provided by 10.13039/501100001691Japan Society for the Promotion of Science. Ai Takemura reports financial support was provided by 10.13039/100017093Yamaha Motor Foundation for Sports. If there are other authors, they declare that they have no known competing financial interests or personal relationships that could have appeared to influence the work reported in this paper.

## Data Availability

Data will be made available on request.
