# Supplementary material for: Mild hyperbaric oxygen does not attenuate mitochondrial decrease induced by detraining in mice
Source: Biochem Biophys Rep. 2025 Nov 6;44:102341. doi: 10.1016/j.bbrep.2025.102341 (PMC12639397; doi:10.1016/j.bbrep.2025.102341)
Supplement: Multimedia component 1 [file mmc1.pptx]

## Slide 1
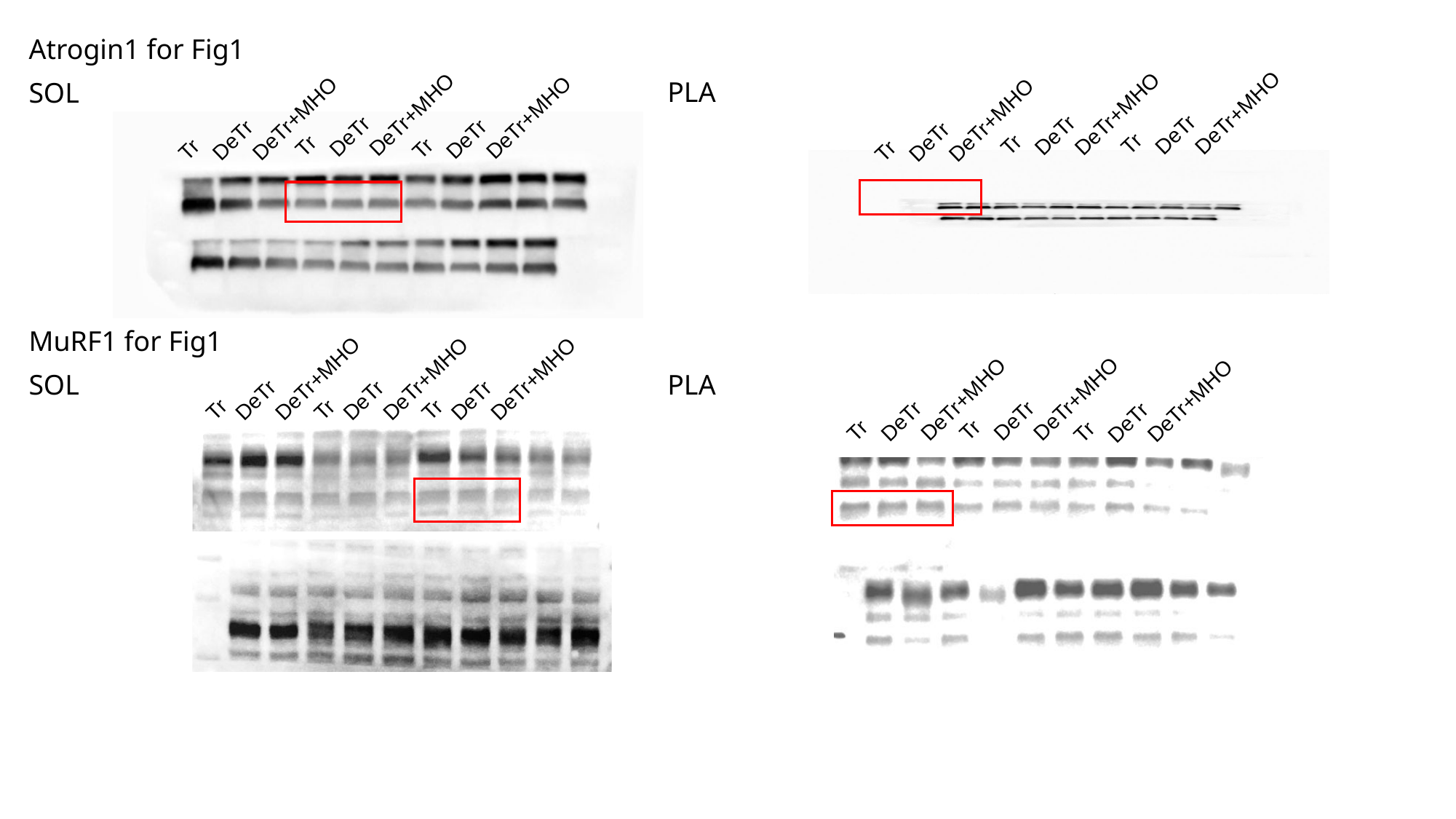

Atrogin1 for Fig1
PLA
SOL
DeTr+MHO
DeTr+MHO
DeTr+MHO
DeTr+MHO
DeTr+MHO
DeTr+MHO
DeTr
DeTr
DeTr
DeTr
DeTr
Tr
Tr
DeTr
Tr
Tr
Tr
Tr
MuRF1 for Fig1
DeTr+MHO
DeTr+MHO
DeTr+MHO
PLA
SOL
DeTr+MHO
DeTr+MHO
DeTr+MHO
DeTr
DeTr
DeTr
Tr
Tr
Tr
DeTr
DeTr
DeTr
Tr
Tr
Tr

## Slide 2
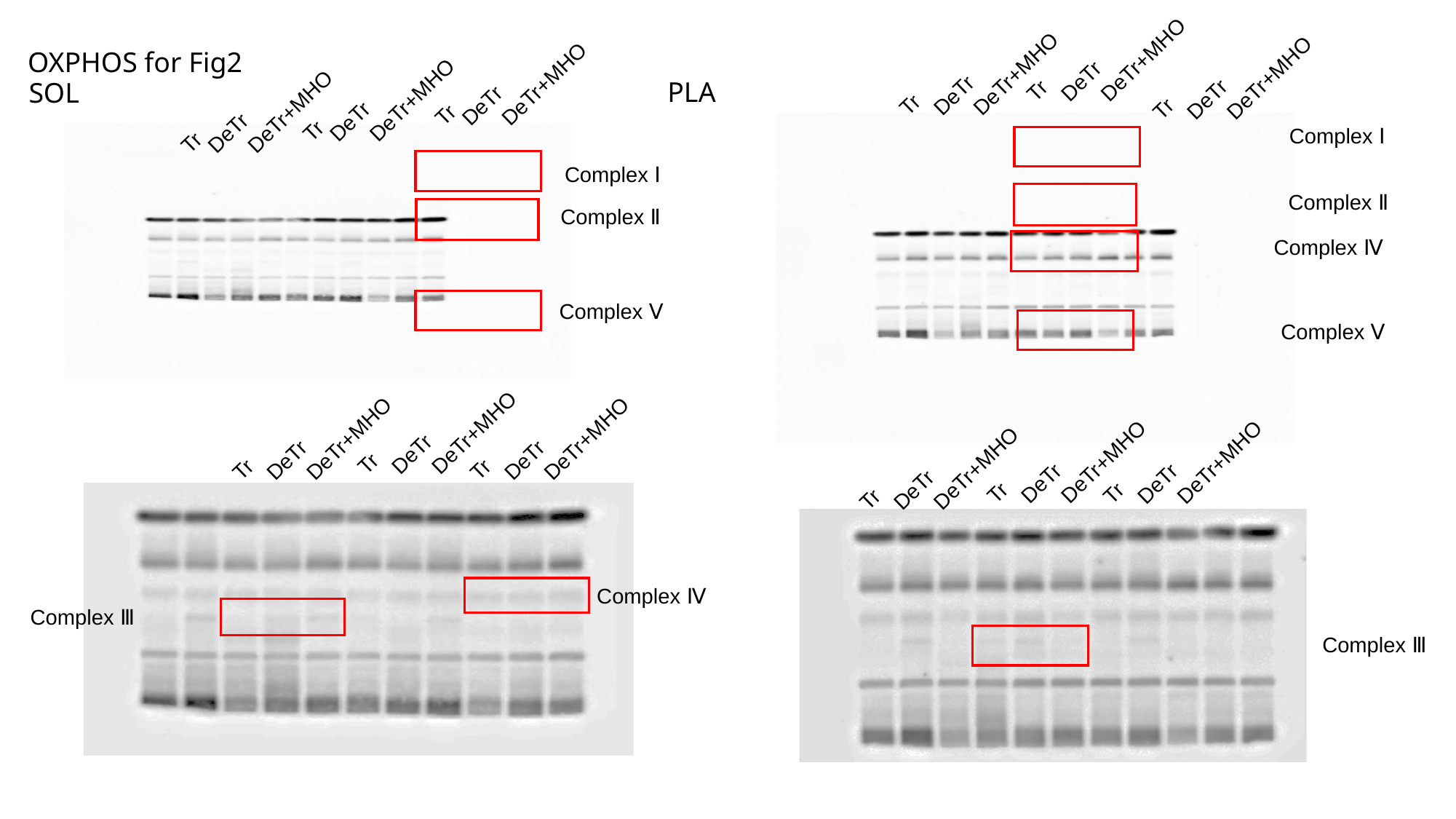

DeTr+MHO
OXPHOS for Fig2
DeTr+MHO
DeTr+MHO
DeTr
DeTr+MHO
Tr
PLA
SOL
DeTr
DeTr+MHO
DeTr
Tr
Tr
DeTr
DeTr+MHO
Tr
DeTr
Tr
DeTr
Complex Ⅰ
Tr
Complex Ⅰ
Complex Ⅱ
Complex Ⅱ
Complex Ⅳ
Complex Ⅴ
Complex Ⅴ
DeTr+MHO
DeTr+MHO
DeTr+MHO
DeTr
DeTr
DeTr
Tr
DeTr+MHO
DeTr+MHO
Tr
Tr
DeTr+MHO
DeTr
DeTr
Tr
DeTr
Tr
Tr
Complex Ⅳ
Complex Ⅲ
Complex Ⅲ

## Slide 3
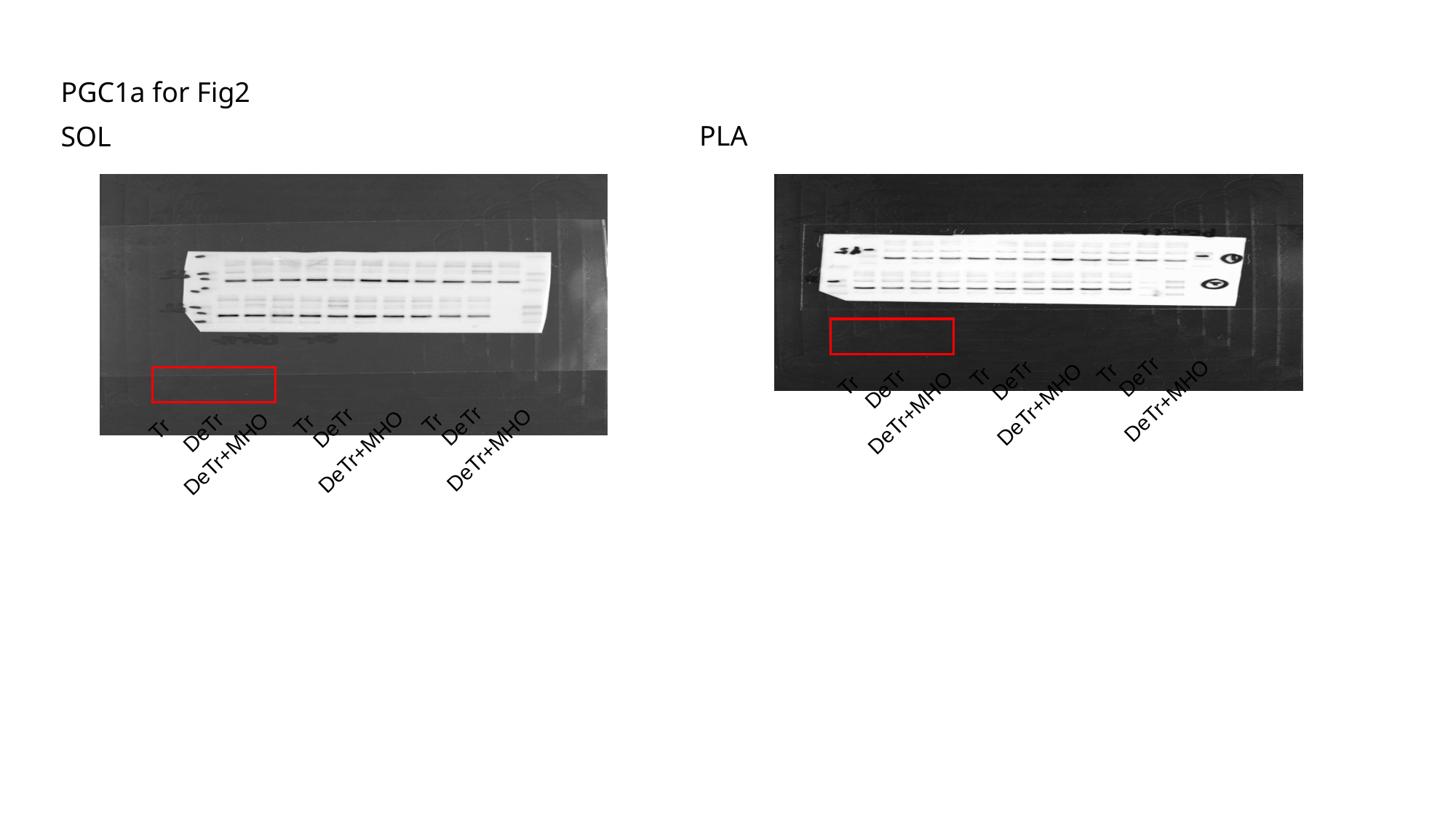

PGC1a for Fig2
PLA
SOL
Tr
Tr
DeTr
DeTr
Tr
DeTr
DeTr+MHO
DeTr+MHO
DeTr+MHO
Tr
Tr
DeTr
Tr
DeTr
DeTr
DeTr+MHO
DeTr+MHO
DeTr+MHO

## Slide 4
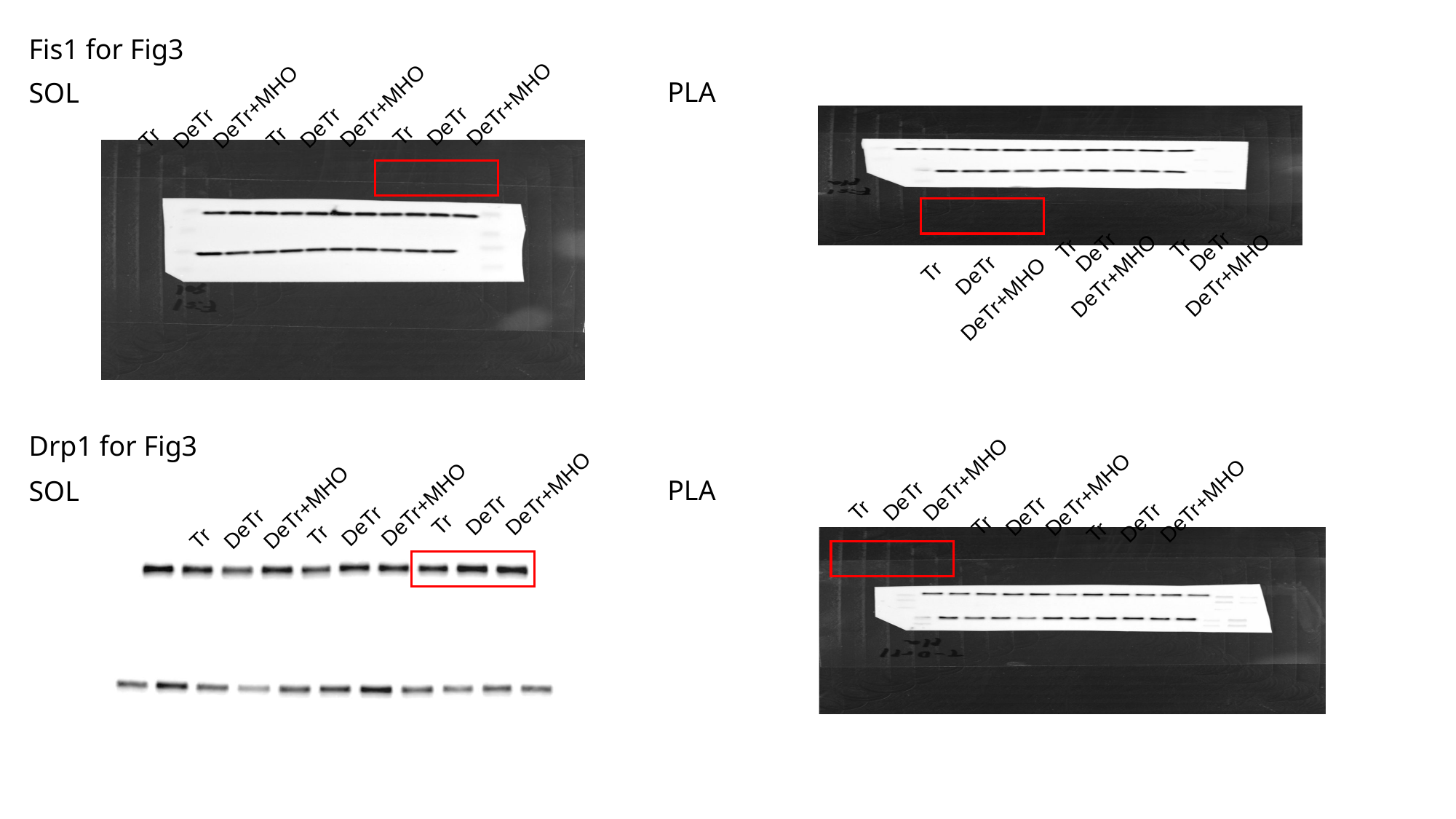

Fis1 for Fig3
PLA
SOL
DeTr+MHO
DeTr+MHO
DeTr+MHO
DeTr
DeTr
DeTr
Tr
Tr
Tr
Tr
Tr
DeTr
DeTr
Tr
DeTr+MHO
DeTr+MHO
DeTr
DeTr+MHO
Drp1 for Fig3
DeTr+MHO
PLA
SOL
DeTr+MHO
DeTr+MHO
DeTr+MHO
DeTr
DeTr+MHO
DeTr+MHO
Tr
DeTr
DeTr
Tr
DeTr
Tr
DeTr
Tr
DeTr
Tr
Tr

## Slide 5
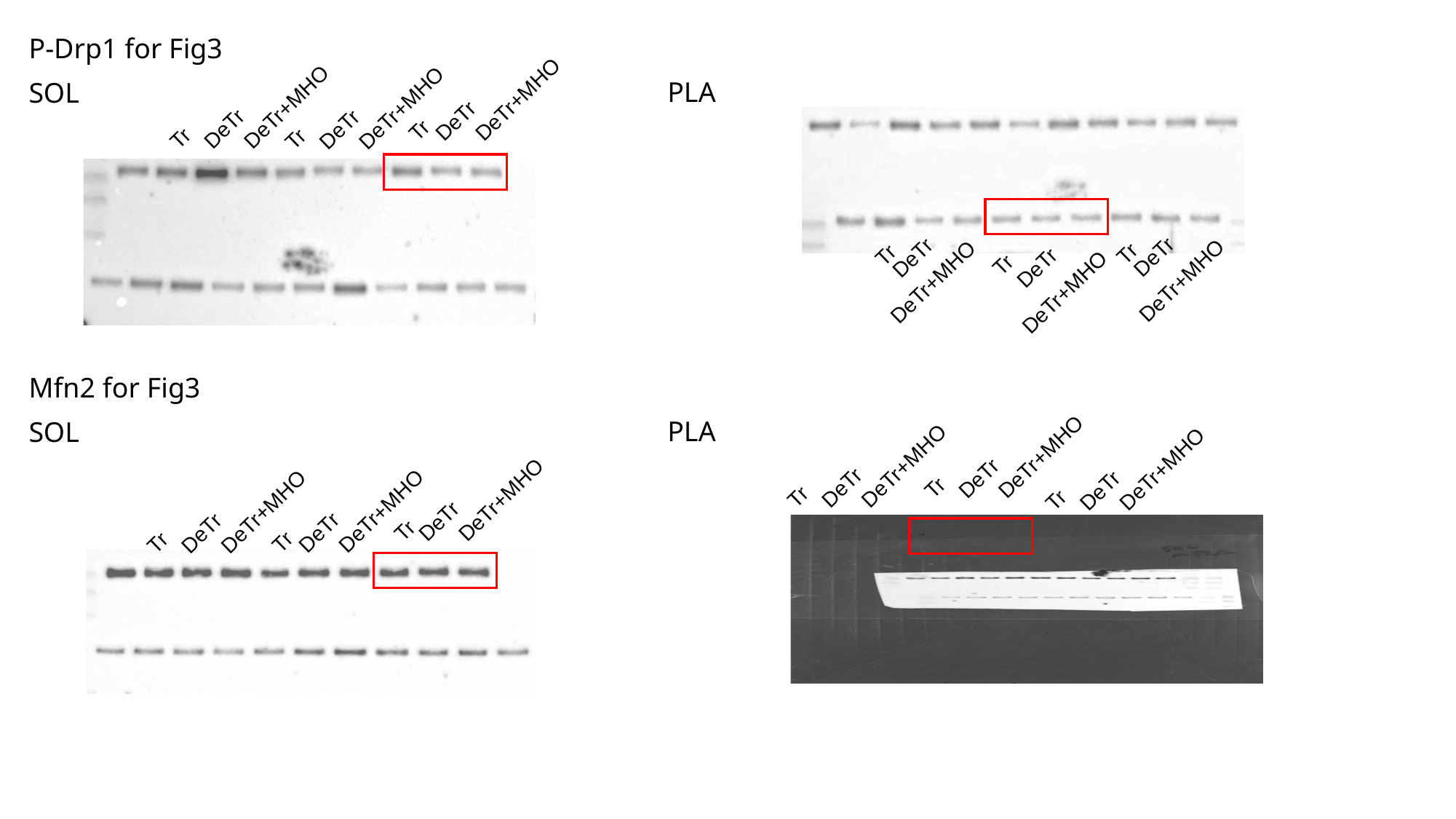

P-Drp1 for Fig3
PLA
SOL
DeTr+MHO
DeTr+MHO
DeTr+MHO
DeTr
Tr
DeTr
DeTr
Tr
Tr
Tr
Tr
DeTr
DeTr
Tr
DeTr
DeTr+MHO
DeTr+MHO
DeTr+MHO
Mfn2 for Fig3
PLA
SOL
DeTr+MHO
DeTr+MHO
DeTr+MHO
DeTr
Tr
DeTr
DeTr
Tr
Tr
DeTr+MHO
DeTr+MHO
DeTr+MHO
DeTr
Tr
DeTr
DeTr
Tr
Tr

## Slide 6
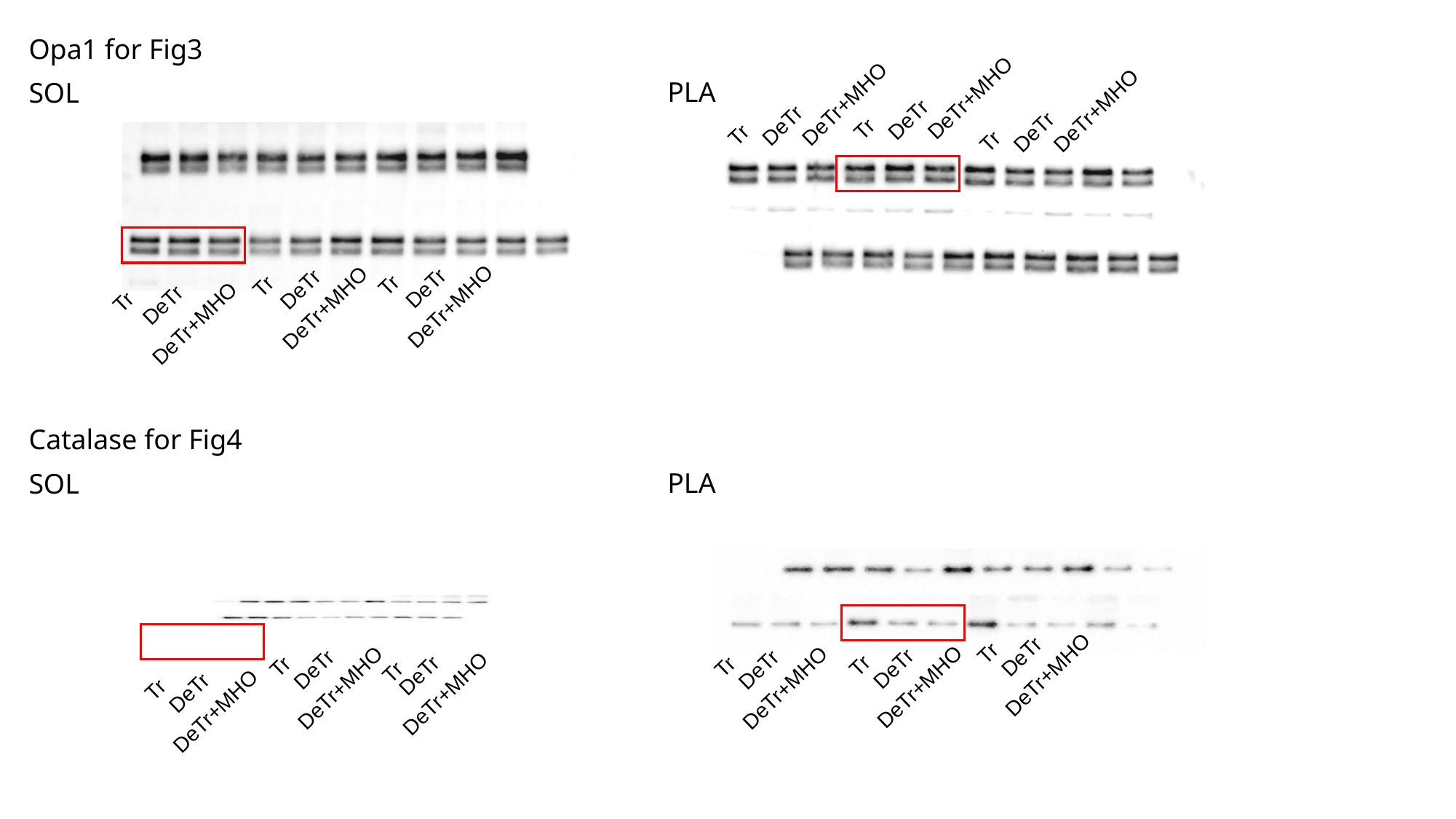

Opa1 for Fig3
PLA
SOL
DeTr+MHO
DeTr+MHO
DeTr+MHO
DeTr
DeTr
Tr
Tr
DeTr
Tr
Tr
Tr
DeTr
DeTr
Tr
DeTr
DeTr+MHO
DeTr+MHO
DeTr+MHO
Catalase for Fig4
PLA
SOL
Tr
DeTr
Tr
Tr
Tr
DeTr
Tr
DeTr
DeTr
DeTr+MHO
DeTr
DeTr+MHO
Tr
DeTr+MHO
DeTr+MHO
DeTr+MHO
DeTr
DeTr+MHO

## Slide 7
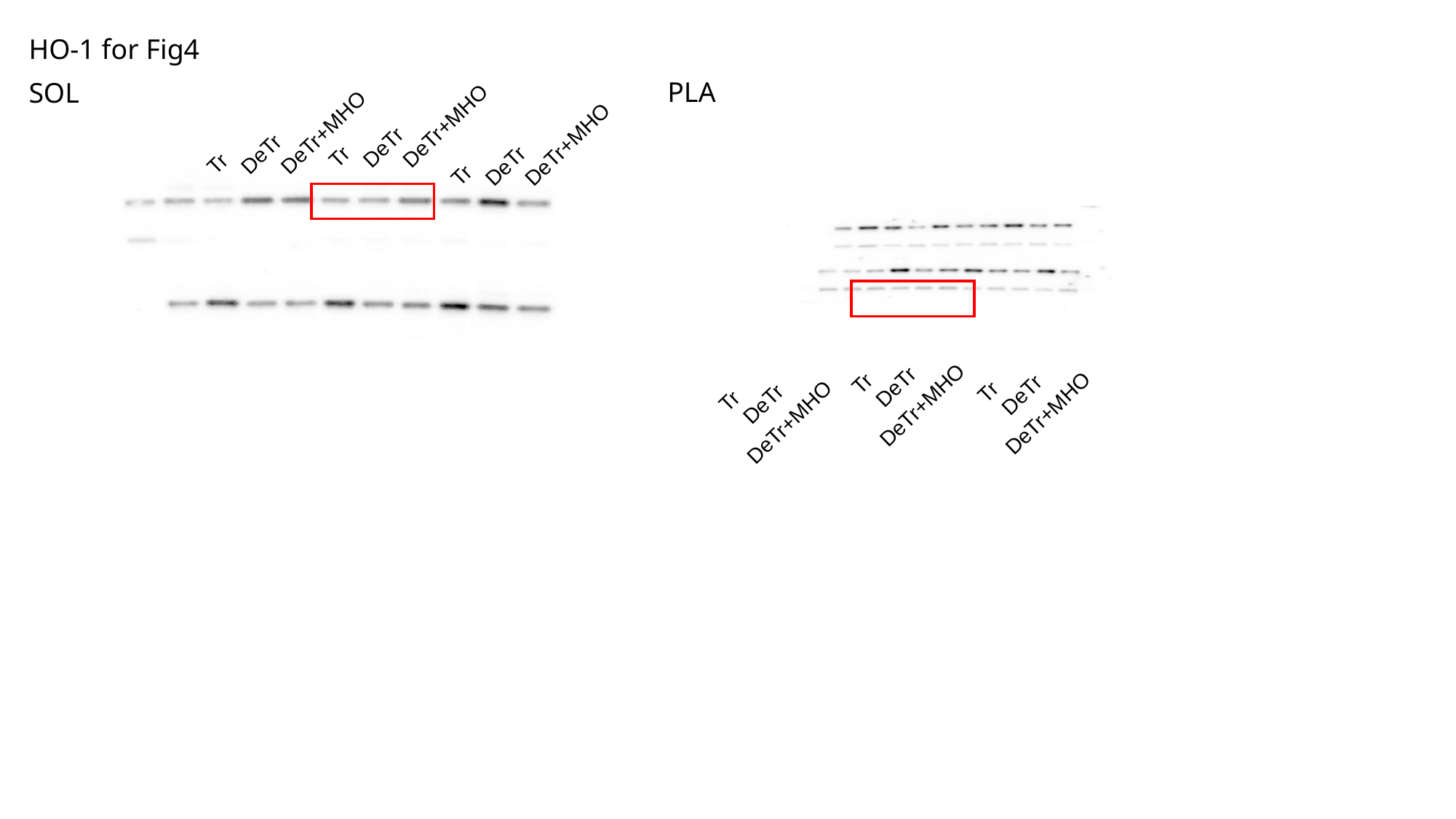

HO-1 for Fig4
PLA
SOL
DeTr+MHO
DeTr+MHO
DeTr+MHO
DeTr
Tr
DeTr
Tr
DeTr
Tr
Tr
DeTr
Tr
DeTr
Tr
DeTr+MHO
DeTr
DeTr+MHO
DeTr+MHO
